# New impulses from international development for more comprehensive and balanced public engagement evaluation

**DOI:** 10.1080/16549716.2019.1680067

**Published:** 2019-11-04

**Authors:** Marco J. Haenssgen

**Affiliations:** Global Sustainable Development, School of Cross-Faculty Studies, University of Warwick, Coventry, UK; Institute of Advanced Study, University of Warwick Science Park, Coventry, UK

**Keywords:** Antimicrobial Resistance, Public engagement, patient and public involvement, participatory research, health research, evaluation, Thailand, Laos

## Abstract

Public engagement in health research has gained popularity because of its potential to co-create knowledge, generate dialogue, and ground research in the priorities and realities of the target groups. However, public engagement that achieves these objectives could still entail unforeseen negative consequences or a wasteful use of resources. Although the evaluation of public engagement has evolved in recent years, we lack consistent evaluation criteria for systematic and transparent assessments of success and failure. This article introduces standard evaluation criteria from the field of development aid evaluation (effectiveness, efficiency, impact, relevance, sustainability) to promote more systematic and comprehensive evaluation practice. I apply these criteria to the public engagement component of a recent research project into antimicrobial resistance, antibiotic use, and health behaviour in Thailand and Laos. Considering village-level engagement workshops, international exhibitions of photo narratives of traditional healing in northern Thailand, and social media communication, I demonstrate that activities that seem to achieve their objectives can still have problematic characteristics in other dimensions. I conclude that these five generic evaluation criteria can broaden our understanding of public engagement. Their more widespread use in evaluations can help build a more comprehensive and balanced evidence base, even if only a sample of public engagement projects and programmes can be evaluated systematically.

## Background

Public engagement remains high on researchers’ and funders’ agendas, and it has particular prominence in health research. The UK Medical Research Council (MRC) advises for instance that, ‘effective public engagement is a key part of the MRC’s mission and all MRC-funded establishments are encouraged to dedicate resources to support this area of work’ and the Wellcome Trust has awarded more than £30 million for dedicated public engagement projects between 2005 and 2018 [1,2].

Also framed as science communication, community engagement, or patient and public involvement in health research, the broad definition of public engagement has evolved from the unidirectional transfer of scientific knowledge from researchers to the lay public, to bidirectional and collaborative ‘engagement’ with the users of research and non-academics more broadly [3–7]. Such engagement aims at broadening the appreciation and impact of research, but collaborative relationships are also intended to improve relevance and ethical aspects of health research and policy – for instance by enabling scientists to learn from their target populations and define and guide research and practice in a participatory fashion [8–12].

In global health research and policy, public engagement activities tend to cluster around the instrumental rather than collaborative end of the spectrum, involving typically behaviour change interventions, health education campaigns, or activities to ‘mobilise’ communities for instance to vaccinate their children [13–17]. The types of activities employed for these purposes include, for example, online information platforms, science festivals and museum events, theatre performances, radio shows, or information workshops for high school students [6,18–21]. Public engagement following the collaborative strand involves longer-term interaction and partnerships such as the establishment of community health committees to advise the local health system and researchers [5,22].

Whether it is framed as a dialogue or as an intervention to educate and mobilise, public engagement can have unintended consequences in spite of its potential benefits and the motivation of researchers. Target and non-target groups can experience negative consequences and outright harms, like misunderstandings, suppression, or stigmatisation. For example, as a form of health communication, public engagement does not only help to expand knowledge and spark curiosity, but it can also create resistance or actions with problematic consequences [23,24]. In Denmark, public awareness raising about drug resistance has eventually culminated in leaflets that advocate not to have sex with pig farmers [25]. In The Gambia [13], radio programmes, theatre performances, and consultative workshops were used to encourage community participation vaccine trials, but these activities eventually reproduced an artificial, dichotomous distinction between ‘accepting’ and ‘refusing’ communities (the idea that people’s participation in the trial should be expected, and that refusal to participate is a problem, caused by ignorance). The failure to enact bidirectional communication cemented misunderstanding, discredited communities’ understanding of the trial, and obscured local struggles of care practices and gender, among others – with uncertain long-term consequences for the participants and their relationships with the local health system, international researchers, and intermediate actors like government staff. Even long-term community engagement through health committees can face challenges like the risk of polarisation within a community or that local elites instrumentalise the committee for political purposes and personal gain [22].

Research funders like the UK Research Councils have kept public engagement high on their impact agendas. Yet, after several years of practice, our understanding of the opportunities and limitations of public participation in research, of its potential costs, and of the various designs that can be employed in different contexts remains limited. Extensive and systematic evaluation could inform these dimensions of public engagement.

Methods for the evaluation of public engagement exist, but their implementation is often subject to varying evaluation criteria (see Section 2 for a brief overview). This means that it is difficult if not impossible to gauge what kind of engagement is effective, where we might see the highest risk of negative consequences (and thus could try to mitigate them), and which of them are, by-and-large, a good use of resources. The lack of knowledge about the outcomes and impacts of engagement activities, projects, and programmes is, in fact, one of the reasons why the Wellcome Trust recently announced that it will discontinue its current public engagement funding model [26].

The purpose of this article is to promote more systematic and transparent assessments of success and failure in public engagement evaluation by introducing a standard approach in international development aid, namely the five evaluation criteria effectiveness, efficiency, impact, relevance, and sustainability [27,28]. (I will not elaborate on evaluation designs and paradigms, data collection, and analysis methods, for which there is a wide literature available [29–32]; nor will I critique the practices of public engagement or evaluation themselves). These criteria – which are best understood as dimensions or categories of assessment, rather than as a method, a model, or a set of indicators – were introduced by the Development Assistance Committee (DAC) of the Organisation for Economic Co-operation and Development (OECD) in 1991 and have since become standard for ex post evaluations of aid projects and programmes. (For example, between 1999 and 2018, German development co-operation conducted 1,794 evaluations of government-supported aid projects and programmes using the DAC criteria according to biannual evaluation reports of the Independent Evaluation Unit of the Kreditanstalt für Wiederaufbau Entwicklungsbank). The role of the DAC has also recently come to the fore in UK health research: as the Global Challenges Research Fund supports international research as part of the UK aid budget, it aims to benefit especially countries that are categorised as middle income or below according to the OECD DAC classification. This development offers an opportunity to link aid and health research also with respect to evaluation practice.

In the remainder of this article, I will provide a brief overview of current evaluation approaches for public engagement before introducing the DAC evaluation criteria (translated to the specific setting of public engagement). To illustrate how the criteria can be used to stimulate a balanced assessment, I will describe a case study of social research in Thailand and Lao PDR on antimicrobial resistance [one of the top 10 global health topics 2019 named by the World Health Organization; 33]. The case involves a range of engagement activities that fall in the middle of the instrumental–collaborative spectrum, including workshops, photo exhibitions, and social media outreach. This analysis will demonstrate that merely achieving the objectives of an engagement activity does not automatically mean that it was successful. I conclude with a call for a more systematic evaluation of public engagement to better understand how (not) and under which circumstances (not) to engage.

### Current practice in evaluating public engagement

Public engagement depends and thrives on creativity to make scientific concepts and methods more widely accessible, and to enable researchers to learn from target groups on equal terms. This creates similarities between evaluating public engagement with research, evaluating artistic projects (e.g. exhibitions or film projects), and evaluating participatory research (e.g. community-based research partnerships) [34,35]. Despite the growing role of public engagement in health research, guidance and methods to evaluate its effectiveness have developed comparatively slowly [10,36], and evaluation techniques for creative activities and participatory research are especially limited although their challenges and risks are recognised [36–40].

In general, different evaluation approaches include process, ex post, and impact evaluations; which may or may not be theory driven (e.g. realist evaluation); which can utilise quantitative, qualitative, or a combination of these methods; and employ specific evaluation frameworks and assessment tools. For these general topics, the interested reader may consult for instance a review of the use of evaluation tools in health-related public engagement by Boivin *et al*. [8]; a detailed introduction to evaluation methods in the context of science education by Friedman [32]; and more general texts on qualitative, quantitative, and interdisciplinary evaluation by Bell and Aggleton [41]. Among recent contributions is also the extensive evaluation method library of The Global Health Network [31], which provides public-engagement-specific resources for instance on realist evaluation, theory of change, or evaluating social media engagement.

For the purposes of this article, I focus specifically on overarching evaluation criteria (rather than other aspects of evaluation), which I illustrate with three examples below. Firstly, in the context of public engagement with science, Rowe *et al*. [42] present an evaluation case study of the 2003 public debate ‘GM Nation?’ about genetically modified food crops. The authors suggest three evaluation criteria, by which they refer to engagement objectives and goal indicators from the perspectives of three different groups of actors: sponsors (e.g. to let the public frame the issue), participants (e.g. whether they learned something from the debate), and academia (e.g. the activity to have an impact on policy). However, the authors also concede that their evaluation criteria selection relates primarily to assessing effectiveness.

As an example of evaluating participatory research, Holkup *et al*. [40] review a study on health inequities with a Native American community in the US. The authors guide their evaluation by an adaptation of quality criteria for qualitative research and naturalistic enquiry (e.g. the equivalent of ‘replicability’ for quantitative research). Yet, the authors note the limitations of this approach, which for instance does not correspond to requirements of long-term viability of participatory action beyond the scope of a discrete project. The ensuing list therefore includes the four criteria ‘level of community involvement,’ ‘community voice,’ ‘acceptable problem resolution,’ and ‘feasibility of project sustainability’ – plus the informal requirement to maintain cohesion among the team of researchers with diverse viewpoints.

With respect to evaluating creative forms of public engagement with health research, Austen [19] summarises findings from the 2016 Wellcome Trust International Engagement workshop on ‘The Art of Health.’ Central themes of the workshop were the conflicting agendas of artists, scientists, and funders when considering the objectives of creative collaborations, the role of important yet unquantifiable creative processes, the unpredictable nature of artistic production, and the long time span during which impacts might materialise. A project in which such considerations materialised was the artist-led and UK-hospital-based 2015/2016 exhibition ‘Under the Microscope,’ which catered especially to paediatric patients [39]. The qualitative evaluation (using thematic analysis of interviews and feedback questionnaires) focused on project management and the achievement of tangible targets like visitor numbers alongside intangible objectives like the provision of a creative framework and artistic vocabulary to mediate relationships between patients and clinicians.

These three examples are representative for the wide range of explicit or implicit criteria according to which a public engagement project is judged successful. Where a formal evaluation process is pursued, evaluations in public engagement typically start with a project-specific list of criteria, most of which can be subsumed under the generic heading of ‘effectiveness’ as goal achievement, with occasional extensions to other areas like long-term viability. This is not to say that these examples have not produced valuable insights in their own right. However, the variability of these evaluations is problematic because it obscures what exactly is being evaluated – a shortcoming that becomes visible in the light of the several decades of evaluation experience in fields like international development. As an alternative, I propose the application of the DAC criteria as the basis for formulating project-specific indicators and targets. I explain the five generic DAC criteria and translate them into the context of public engagement in the following section.

### Five criteria for evaluating public engagement projects

The criteria of effectiveness, efficiency, impact, relevance, and sustainability were devised originally for aid projects, but they can be applied usefully to public engagement as well (as my own evaluation experience for instance with arts-based engagement has shown). This section introduces each criterion with its DAC definition [28], after which I apply it to public engagement. In addition to these five generic criteria, aid evaluation practice has over the years also increasingly recognised issues like representation, equity, and gender, which influence the judgement of success as cross-cutting concerns rather than as alone-standing criteria [43].

**Effectiveness**: ‘A measure of the extent to which an aid activity attains its objectives’ [28]. Effectiveness assesses whether and to what extent the public engagement objectives have been achieved. The objectives are chosen by the public engagement project itself rather than by the evaluator. In addition, they need not only focus on target group outcomes, but could also include for instance fostering collaboration, gaining new insights for research, or promoting non-academic engagement (e.g. with political processes, with the arts and crafts, or within participants’ local communities).

**Efficiency**: ‘measures the outputs – qualitative and quantitative – in relation to the inputs’ [28]. Efficiency can be divided into three parts. First, production efficiency would assess if the engagement activity complied with its timeline, whether resources were used appropriately, and whether the target group was reached as planned. Second, allocative efficiency of the project assesses whether resources could have been spent more usefully to achieve the engagement goals. Third, cost-effectiveness assesses the total costs of developing and delivering the activities relative to outputs, outcomes, and impacts. On the output level, this would for instance be the population reached per £ spent. However, the cost of outreach is a very crude indicator for the efficient use of resources. Evaluators would preferably assess whether the resources were effective in actually ‘engaging’ the target audiences. Information about lasting effects beyond the activity itself – for example, participants sharing their experiences and inspiration with their families – would enable yet more detailed (and potentially more favourable) assessments of cost-effectiveness. Different target groups may also be harder to reach or to engage with, which may be reflected in the cost-effectiveness of the project. Qualitative information is therefore helpful to contextualise cost-effectiveness figures.

**Impact**: ‘The positive and negative changes produced by a development intervention, directly or indirectly, intended or unintended’ [28]. To enable an assessment of impacts, the evaluation needs to include methods that are able to capture side-effects alongside the primary objectives. Larger-scale programmes may thereby relate even to societal-level impacts like mortality or enrolment rates, while the impact on a smaller scale could include for instance the formation of a research team comprising members of the public and academics who bid together for project funding. Across all kinds of impact, equity effects are also important to consider – for instance, gender dimensions or whether the activity reproduces existing forms of inequality and discrimination.

**Relevance**: ‘The extent to which the aid activity is suited to the priorities and policies of the target group, recipient and donor’ [28]. Relevance considers whether the objectives of the activity correspond to target group requirements, but also to national and global priorities as well as partners’ and – in some cases – donors’ policies. Importance for the researcher does not automatically imply relevance of the project for the target groups. Relevance also addresses whether the public engagement activity suggested a plausible mechanism to achieve its objectives, and whether it aligned with and/or integrated into parallel engagement activities.

**Sustainability**: ‘is concerned with measuring whether the benefits of an activity are likely to continue after donor funding has been withdrawn. Projects need to be environmentally as well as financially sustainable’ [28]. Sustainability considers the public engagement activity from a long-term perspective, especially whether its effects and impacts would persist beyond the end of the activity. This is a particular challenge in isolated projects, especially if they do not align with other forms of public engagement. One way to assess sustainability in discrete project could be for instance to gauge after several months whether participants retain details of events, whether changed behaviours persist or revert to their previous state, or whether newly formed relationships last.

## Case study: applying the evaluation criteria to public engagement with research

### Overview

I will illustrate the application of these generic evaluation criteria with the case of the public engagement activities of the ‘Antibiotics and Activity Spaces’ project [27]. The research project took place from 2017 to 2018 and studied health behaviour and antibiotic use in rural Thailand (Chiang Rai province) and Lao PDR (Salavan province) against the thematic backdrop of antimicrobial resistance (AMR; also ‘drug resistance’ or ‘drug-resistant bacteria’). The project arose in response to the problematic dominance of awareness campaigns in global health policies as primary means to tackle drug resistance among the general population. Through a large rural survey supplemented with interviews, the research team investigated (1) treatment-seeking pathways in the general population to identify which behaviours could be deemed ‘problematic’ (e.g. forms of medicine use that are likely contribute to drug resistance); (2) the levels, spread, and behavioural consequences of knowledge about antibiotics and drug resistance; (3) and indicators to detect potentially problematic behaviours.

### Public engagement activities

The research project involved several forms of public engagement, which were carried out between 2017 and early 2019. Firstly, the survey teams hosted half-day workshops in 2017 in all five census survey villages [described in detail in 44, 45]. The half-day workshops catered to 20 to 35 adult participants and intended to facilitate bi-directional knowledge exchange between residents and the research team through activities like community mapping, categorisation of common medicines through pile sorting, drug-resistance-themed games and role-plays, and poster making to feedback participants’ workshop interpretations to the study team. The workshop content was developed by Southeast Asian research team members with experience in interdisciplinary and social sciences AMR research as well as in community-based development.

Secondly, the public engagement activities involved the collection and exhibition of photographic narratives of traditional treatment (the narratives were collected in a subset of 10 villages in Chiang Rai, covering both main survey and census survey villages; see next sub-section for an overview of project activities). The activity arose from the feedback of the Thai survey team, who reported that the treatment-seeking behaviour questionnaire did not capture important aspects of local traditional healing such as herbal medicine and summoning ghosts. Upon request of the research team, the villagers permitted us to document and exhibit their stories of healing – which we did in the ‘Tales of Treatment’ photo exhibition series in Bangkok (Art Gallery g23), Chiang Rai (Tai tea shop and bar), Oxford (Green Templeton College), and Coventry (Warwick Arts Centre) between July 2018 and March 2019 (https://tinyurl.com/talesoftreatment). The exhibition was curated by the Thai research project officer Nutcha Charoenboon together with the project team members Patthanan Thavethanutthanawin and Kanokporn Wibunjak.

Thirdly, the project placed emphasis on reaching wider non-academic audiences through social and traditional media – in Thailand, Lao PDR, UK, but also in other countries. Outreach took place continually alongside the research and public engagement activities, especially on the platforms *Facebook, Twitter, LinkedIn*, and *Reddit*. This activity was directed by the author.

Taken together, the objectives of these public engagement activities were to (1) share information with the village communities about drug resistance and local forms of treatment in our research sites (not primarily to change people’s behaviour) (workshops), to (2) learn from our participants about their medicine use and health behaviours (workshops, exhibition), and to (3) acquaint a broad range of the non-academic public in Thailand, Lao PDR, UK, and beyond with our research to increase the interest in social studies of antibiotic resistance (exhibition, social media).

In the following illustrative application of the evaluation criteria, I will jointly consider all three types of engagement activities.

### Data

Data to support the illustration were drawn from the research project and the public engagement activities, comprising surveys, interviews, observations, and oral and written feedback.

**Figure 1.**
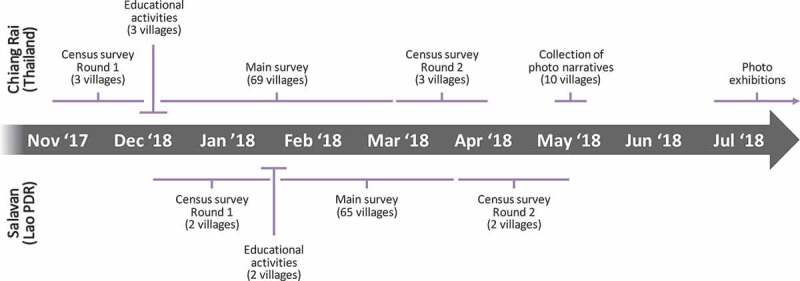
Timeline of project activities

The survey was the main component of the research project (see Figure 1 for a timeline of project activities). The survey had two parts to enable representative provincial-level as well as in-depth community-level analysis of health behaviour in the rural field sites: One part was a cross-sectional systematic random survey, designed to be representative for the rural populations of Chiang Rai and Salavan (2,141 responses across 134 villages representing approx. 712,000 villagers). The second part was a two-round census survey of all adult residents in three villages in Chiang Rai and two villages in Salavan, taking place in an interval of 3 months (3,744 responses). The aforementioned public engagement workshops took place in between these two rounds (depicted in Figure 1 together with the survey activities and the collection of photo narratives). The surveys used a 45-minute health behaviour questionnaire that was developed based on prior qualitative research on treatment seeking and antibiotic use in Southeast Asia [46], comprising modules on socio-demographic background, knowledge and attitudes towards antibiotics and drug resistance, and the step-by-step process of seeking care for an acute illness or discomfort experienced by the respondent or a child under their supervision. Situating the workshops in between the survey rounds enabled a short-term assessment of knowledge, attitude, and behaviour changes among participants and non-participants in the five census survey villages. The survey teams comprised six to eight field investigators plus two supervisors in each of the two sites.

In addition, we conducted 50 cognitive interviews prior to and alongside the survey [47]. Cognitive interviews are a semi-structured interview technique in which specific survey questions such as the treatment-seeking process or the knowledge of antibiotics are examined in detail to understand the respondent’s thought process and the context in which they arrive at their answers. However, aside from the primary purpose of questionnaire development, the qualitative data generated through this method also supplied contextualising information for the interpretation of the survey results and the behavioural consequences of the public engagement workshops. The cognitive interviews were conducted by the survey team supervisors within and jointly across the two sites.

Alongside the engagement activities (workshops and exhibition), we collected further data that supported the informal evaluation. On the one hand, non-participant observations were recorded from the engagement workshops and from regular survey review meetings (of which the participants were aware). On the other hand, participants of the exhibitions shared oral and written feedback with the permission to use the materials for evaluation purposes. Evaluation feedback forms were distributed at one of the venues (Warwick Arts Centre), for which we achieved a response rate of 23 out of 70 attendees (33%).

Although we did not conduct a formal evaluation (prevented by our position as project insiders and lacking funds for an external evaluation), the varied sources enable at least an informal and illustrative short-term review of our activities in terms of effectiveness, efficiency, relevance, impact, and sustainability.

### Effectiveness

In terms of goal achievement, Objective 1 focused on sharing information about drug resistance with the village communities where we hosted the workshops. The survey data demonstrated that workshop participants had a 30 percentage-point higher awareness about drug resistance after the event, compared to an increase of 17 percentage points in the villages more generally [44,45]. This indicates that, at least on the face of it, Objective 1 had been achieved.

With respect to Objective 2 (learn from participants about their medicine use and health behaviours), the workshops and photographic narratives enabled us to reflect on the relationship between traditional healing and drug resistance. Among the stories narrated in ‘Tales of Treatment’ is, for instance, a Mien village in Chiang Rai where spiritual healing is practised. Although a spiritual healer (‘ghost doctor’) has inherited and mastered a sacred book of elaborate healing ceremonies, the village residents themselves can (and routinely do) perform small ceremonies on their own. This story is one among several that have challenged our team’s initial conceptions and understanding of healing and treatment in rural Chiang Rai – in this case, our initially dichotomous conception of ‘traditional healer’ and ‘resident.’ In addition, the workshops allowed the project team to formulate and test hypotheses about antibiotics use that would have otherwise remained invisible. For example, participants categorised different types of antibiotics into the groups ‘you can buy this medicine over the counter’ and ‘you need a prescription from a doctor to obtain this medicine.’ While the survey did not anticipate this category, our survey data indicated that people’s attitudes regarding antibiotics differed substantially depending on how they referred to the medicine – whereby people using technical language to describe antibiotics were more aligned with the attitudes that the World Health Organisation recommends (i.e. not to buy antibiotics over the counter; the analysis is described in more detail in a forthcoming publication). Both quantitative data and reflective accounts suggest that Objective 2 was achieved as well.

We achieved our third objective less clearly (acquaint the broader public with our research). As described further in ‘Efficiency’ below, the exhibitions hosted 500 visitors who engaged with the content enthusiastically and expressed a strong interest in the subject. The feedback forms collected at the Warwick Arts Centre also suggested that the respondents overwhelmingly agreed that they ‘learned something new’ (95.7% of 23 respondents). However, the social media activities were less clear in their effective outreach. Although we reached people superficially through several hundred thousand ‘impressions’ and more than ten thousand post ‘engagements’ internationally (e.g. shares, ‘likes,’ link clicks), we received virtually no direct feedback or follow-up from non-academic audiences to our social and mass media campaigns.

### Efficiency

Basic efficiency assessments can be made for all three types of engagement activities. In terms of outreach, the workshops involved a total of 150 participants from five villages with a total population of approximately 2,000 people. The photo exhibitions involved the collection of 15 narratives, which were presented to approximately 500 visitors across four locations (Bangkok, Chiang Rai, Oxford, Coventry), and an online exhibition booklet containing all photographic narratives was viewed by 214 people by 9 June 2019. As part of the project’s communication activities, we also produced infographics, press releases, and had an active social media presence. Between September 2017 and March 2019, we reached 18,500 users with 6,700 post engagements on *Facebook*; 323,000 impressions and 6,200 tweet engagements on *Twitter*; and <10,000 impressions and engagements on *LinkedIn* and *Reddit*.

The costs of these activities varied. Social media activity was free of charge but supplemented with promotion campaigns (approx. £300 in total). The village workshops had a total cost of approximately £2,250 for delivery, plus approximately £3,000 of staff time and consumables for the development and piloting of the content (£5,250 in total). The exhibition involved approximately £5,000 for hosting the four events plus £3,000 for gathering the narratives and developing the content and media (£8,000 in total). All content and activities were produced in-house by the research team and volunteers under their supervision, which gave us maximum control over the process and helped minimise the costs compared to external media, content, and event production.

A simple yet crude measure of cost-effectiveness is the cost per participant: £35 per workshop participant (£15 variable costs plus £20 fixed costs for development), £16 per exhibition visitor (or £11 if the online consumption of the stories is included), and £0.85 per 1,000 social media impressions. On the outcome level, cost-effectiveness is more difficult to assess. For the workshops, a possible indicator could be the costs per ‘more aware’ workshop participant. The most optimistic estimate would include all villagers whose awareness increased (assuming that this was wholly due to the workshop and subsequent word-of-mouth). The most conservative estimate would only include the relative increase among the workshop participants (assuming that awareness would have increased also without the workshop). Possible beneficiary numbers therefore range from 20 to 340, with corresponding costs of £263 to £15 per ‘more aware’ villager. For social media, the costs per ‘engagement’ were £0.02. No engagement figures are available for the exhibitions, but visitors spent on average 30–45 min at the venues and had extensive interactions with the team members.

### Impact

Downstream consequences of our engagement activities could include inspiration to contribute to research and knowledge generation, institutionalised partnerships, or behavioural change with an impact on antimicrobial resistance and mortality. We did not incorporate impact measurements into the assessment for the exhibitions and the social media campaigns. However, we could argue that one indicator of impact would be independent conversations about the research topic on social media that had not been initiated by our team. We did not detect any such online content. On a smaller scale, participants’ feedback indicated heightened interest in traditional medicine, but a potential problematic side-effect was that some participants pointed out that they ‘*never realised how effective these* [traditional] *treatments can be*’ – despite explicit statements from the project team that this was not the intention of the exhibition [48]. However, the lack of follow-up data makes it speculative whether participants’ behaviour indeed changed.

In contrast, survey and interview data collected alongside the village-level workshops offered an opportunity to measure changes in health behaviour and medicine use and the flow of information within the villages [44,45]. The short-term impact within 3 months was mixed, however. The survey data indicated that participants were more likely after the workshops to access healthcare and use medicine in a way that the World Health Organization recommends – for example, not buying antibiotics over the counter from local grocery stores without prescription [49]. This could potentially decelerate the development of drug resistance locally.

However, our research also documented that participants in Salavan reduced their antibiotic use from local shops against a disproportionate increase from public health centres, and non-participating villagers in Chiang Rai increased their antibiotics consumption from local grocery stores. One contributing factor was that a workshop participant in Chiang Rai felt sufficiently informed about antibiotics to start selling them in her store. Such behaviours could potentially also contribute to drug resistance and ill health.

From an equity perspective, the exhibition and online content were primarily consumed by urban and international populations with higher levels of formal education. Even information about the workshop content only circulated in more privileged circles, that is, among villagers with more formal education and wealth [44,45]. Despite active attempts to be inclusive, the distribution of information was therefore not especially equitable.

### Relevance

Drug resistance is a health priority globally and Southeast Asia is a focal region with widespread antibiotic use, increasing rates of resistance, and with busy regional and international travel that can contribute to the global spread of drug-resistant bacteria [50–52]. Nationally, drug resistance is salient in Thailand’s health policy, which has become an international example for responses to AMR [53]. In Lao PDR, AMR has received little though growing attention as it has been recognised in the *Health Sector Reform Strategy and Framework till 2025* and in the formation of a national branch of the Global Antibiotic Resistance Partnership [54,55]. While these developments underline the global and national health policy relevance of AMR in Southeast Asia, it is less obviously a priority issue for rural populations who often face several other livelihood challenges like volatile incomes, discrimination, or exposure to environmental risks.

Furthermore, we hypothesised that the main mechanism leading to behavioural impact was the exchange of ideas and information. However, our survey data analysis in Charoenboon *et al*. [44] showed that knowledge exchange appeared to play less of a role for people’s behaviour than social cohesion: When patients involved another person in their illness (for instance to look after them or drive them to a hospital), their behaviour appeared to be more in line with recommendations of the World Health Organization (this appeared to happen irrespective of the workshops, which did not change social relationships in the villages). Behavioural sciences research more broadly suggests that knowledge, information, and reflective motivation only play a minor role among other drivers of human behaviour [56]. These points suggest that future projects should address broader behavioural pathways to mitigate unintended negative consequences.

### Sustainability

As an isolated research project with a short-term assessment of outputs and impacts, the engagement activities cannot claim sustainable outcomes. Although the content from our activities will continue to be available online, and despite evidence of potentially lasting collaborations, for example, with the performing arts, we have no basis to assert that our attendees and workshop participants will retain workshop and exhibition content over the long term, or that any change in behaviour will last.

## Conclusion

Goal achievement should only be one evaluation criterion along efficiency, relevance, impact, and sustainability. I argued that this insight from development aid evaluation can add depth and balance to an assessment of public engagement. The engagement activities presented in the case study appeared to achieve their objectives, but this did not automatically make them a success: Outreach was wide but limitedly equitable, behavioural impacts on the target populations were partly negative, the project could have considered social pathways to behavioural impact, and the isolated engagement activities were unlikely to be sustainable. Yet, as part of a pump-priming research project, this assessment does not automatically render the activities a failure. The outputs from this research also help to contribute to the empirical knowledge (e.g. as an example of the limits of engagement through communication activities), to methodological evolution (e.g. using community-wide, individual-level survey data to assess behavioural change), and to the transparency of public engagement evaluation (e.g. the explicit consideration of project costs and cost-effectiveness).

What do we learn from reflections on these broader evaluation criteria? For example, health-related public engagement with the general population could be harmonised with and integrated into school-based educational programmes or community development to improve its relevance and sustainability. Preliminary research and workshops could also improve relevance by identifying the livelihood challenges of target groups in low- and middle-income countries and by tailoring engagement activities more directly to their priorities. In addition, future public engagement projects should explore and articulate the mechanisms that lead to the expected outcomes, recognising potentially detrimental impacts.

To establish such arguments more firmly, there are few alternatives to performing more evaluations (and reporting them transparently). Yet, researchers should not attempt a formal evaluation of their own engagement activities owing to their lack of independence. Self-evaluations are prone to emphasising the enjoyment experienced by target groups and the achievement of primary goals. But what might be the behavioural consequences of our actions? Are we reproducing social and economic divisions in the way we reach our target groups? Will the effects of our initiatives last?

In the short term, funders and academic institutions can play an important role in helping researchers coordinate engagement activities and provide signposting to similar projects to maximise complementarities and avoid a potentially patchy and contradictory engagement landscape. In the medium term, funders or independent organisations could contribute teams of experienced external evaluators to accompany public engagement projects from the design phase onwards. This would help to develop a comprehensive knowledge base of the primary outcomes and side-effects of different forms of public engagement across social and geographic contexts – if only on a sample of projects. While these evaluations should be independent, researchers and evaluators could nonetheless work closely together to inform each other, and subsequently co-own the evaluation findings and publish them jointly in order to add to the body of public engagement knowledge.

## Paper context

Public engagement has been heavily promoted in health research, but the lack of consistent evaluation criteria undermines our understanding of its intended and unintended consequences. This paper introduces the generic evaluation criteria of effectiveness, efficiency, impact, relevance, and sustainability and applies them to public engagement. The use of these established concepts can make evaluations more comprehensive and balanced, and ultimately help develop the evidence base of public engagement in global health.

## References

[1] Wellcome Trust Grant funding data 2019; [2019 6 6]. Available from: https://wellcome.ac.uk/funding/people-and-projects/grant-funding-data

[2] Medical Research Council Public engagement funding 2019; [2019 69]. Available from: https://mrc.ukri.org/research/public-engagement/public-engagement-funding/

[3] Leshner AI. Public engagement with science. Science. 2003;299:1. 10.1126/science.299.5609.977 12586907

[4] Retzbach A, Maier M Communicating scientific uncertainty. Commun Res. 2014;42:429–11.

[5] Tindana PO, Singh JA, Tracy CS, et al Grand challenges in global health: community engagement in research in developing countries. PLoS Med. 2007;4:e273. 17850178 10.1371/journal.pmed.0040273 PMC1989740

[6] Hamlyn B, Shanahan M, Lewis H, et al Factors affecting public engagement by researchers: a study on behalf of a consortium of UK public research funders. London: TNS; 2015.

[7] Cargo M, Mercer SL The value and challenges of participatory research: strengthening its practice. Annu Rev Public Health. 2008;29:325–350. PubMed PMID: 18173388. 18173388 10.1146/annurev.publhealth.29.091307.083824

[8] Boivin A, L’Espérance A, Gauvin F-P, et al Patient and public engagement in research and health system decision making: a systematic review of evaluation tools. Health Expectations. 2018;21:1075–1084. 10.1111/hex.12804 PMC6250878 30062858

[9] Staniszewska S, Brett J, Simera I, et al GRIPP2 reporting checklists: tools to improve reporting of patient and public involvement in research. Res Involv Engagem. 2017;3:13. 29062538 10.1186/s40900-017-0062-2 PMC5611595

[10] Lafrenière D, Cox SM ‘If you can call it a poem’: toward a framework for the assessment of arts-based works. Qual Res. 2013;13:318–336.

[11] Kilroy A, Garner C, Parkinson C, et al Towards transformation: exploring the impact of culture, creativity and the arts on health and wellbeing. Manchester: Manchester Metropolitan University; 2007.

[12] Enria L Co-producing knowledge through participatory theatre: reflections on ethnography, empathy and power. Qual Res. 2016;16:319–329.

[13] Fairhead J, Leach M, Small M Public engagement with science? Local understandings of a vaccine trial in the Gambia. J Biosoc Sci. 2005;38:103–116. Epub 11/03. 16266443 10.1017/S0021932005000945

[14] Roh S, Rickard LN, McComas KA, et al Public understanding of one health messages: the role of temporal framing. Public Understanding Sci. 2018;27:185–196. PubMed PMID: 29353551. 10.1177/0963662516670805 29353551

[15] Nyirenda D, Makawa TC, Chapita G, et al Public engagement in Malawi through a health-talk radio programme ‘Umoyo nkukambirana’: a mixed-methods evaluation. Public Understanding Sci. 2018;27:229–242. 10.1177/0963662516656110 PMC5777544 27365364

[16] Lim R, Peto TJ, Tripura R, et al Village drama against malaria. Lancet. 2016;388:2990. 10.1016/S0140-6736(16)32519-3 27998527

[17] Davis M, Whittaker A, Lindgren M, et al Understanding media publics and the antimicrobial resistance crisis. Glob Public Health. 2017;13:1158–1168. 28594309 10.1080/17441692.2017.1336248

[18] Nyirenda D, Makawa TC, Chapita G, et al Public engagement in Malawi through a health-talk radio programme ‘Umoyo nkukambirana’: a mixed-methods evaluation. Public Understanding Sci. 2016;27:229–242. 10.1177/0963662516656110 PMC5777544 27365364

[19] Austen K The art of health: exploring creative engagement with research [workshop report]. Mumbai: Wellcome Trust; 2016.

[20] Allison PD, Higginson P, Martin S Antibiotic resistance awareness: a public engagement approach for all pharmacists. Int J Pharm Pract. 2017;25:93–96. 27443787 10.1111/ijpp.12287

[21] Cohen ER, Masum H, Berndtson K, et al Public engagement on global health challenges. BMC Public Health. 2008;8:168. 18492256 10.1186/1471-2458-8-168 PMC2453523

[22] Abimbola S Beyond positive a priori bias: reframing community engagement in LMICs (epub ahead of print). Health Promot Int. 2019 DOI:10.1093/heapro/daz023 30982066

[23] Pizzo E, Doyle C, Matthews R, et al Patient and public involvement: how much do we spend and what are the benefits? Health Expectations. 2015;18:1918–1926. 24813243 10.1111/hex.12204 PMC5810684

[24] Cho H, Salmon CT Unintended effects of health communication campaigns. J Commun. 2007;57:293–317.

[25] Fynbo L, Jensen CS Antimicrobial stigmatization: public health concerns about conventional pig farming and pig farmers’ experiences with stigmatization. Soc Sci Med. 2018;201:1–8. 29421319 10.1016/j.socscimed.2018.01.036

[26] IVAR Reflections from the review of the wellcome trust’s public engagement fund. London: Institute for Voluntary Action Research; 2019.

[27] Haenssgen MJ, Charoenboon N, Zanello G, et al Antibiotics and activity spaces: protocol of an exploratory study of behaviour, marginalisation, and knowledge diffusion. BMJ Glob Health. 2018;3:e000621. 10.1136/bmjgh-2017-000621 PMC5884330 29629190

[28] OECD Development Assistance Committee Evaluating development co-operation: summary of key norms and standards. Paris: Organisation for Economic Co-operation and Development; 2010.

[29] Forss K An evaluation framework for information, consultation and public participation In: OECD, editor. Evaluating public participation in policy making. Paris: Organisation for Economics Co-Operation and Development; 2005 p. 41–84.

[30] Frewer LJ, Rowe G Evaluating public participation exercises: strategic and practical issues In: OECD, editor. Evaluating public participation in policy making. Paris: Organisation for Economics Co-Operation and Development; 2005 p. 58–108.

[31] The Global Health Network Mesh - community engagement network 2019; [2019 69]. Available from: https://mesh.tghn.org/

[32] Friedman AJ, editor. Framework for evaluating impacts of informal science education projects: report from a National Science Foundation workshop. Alexandria, VA: The National Science Foundation; 2008.

[33] WHO Ten threats to global health in 2019 2019; [2019 9 18]. Available from: World Health Organization Web page: https://www.who.int/emergencies/ten-threats-to-global-health-in-2019

[34] Minkler M Community-based research partnerships: challenges and opportunities. J Urban Health. 2005;82:ii3–ii12. 15888635 10.1093/jurban/jti034 PMC3456439

[35] Langdridge D, Gabb J, Lawson J Art as a pathway to impact: understanding the affective experience of public engagement with film. Sociological Rev. 2019;67:585–601.

[36] Galloway S Theory-based evaluation and the social impact of the arts. Cult Trends. 2009;18:125–148.

[37] Etherton M, Prentki T Drama for change? Prove it! Impact assessment in applied theatre. Res Drama Educ. 2006;11:139–155.

[38] Ledgard A Blood makes noise: evaluation. 2013 [cited 2019 Oct 29] Available from: http://annaledgard.com/wp-content/uploads/BMNReport1.pdf

[39] Ledgard A Under the microscope: evaluation report. 2016 [cited 2019 Oct 29] Available from: http://annaledgard.com/wp-content/uploads/UtMEvalReport-FINAL3.5.16small-1.pdf

[40] Holkup PA, Tripp-Reimer T, Salois EM, et al Community-based participatory research: an approach to intervention research with a Native American community. Adv Nurs Sci. 2004;27:162–175. PubMed PMID: 15455579. 10.1097/00012272-200407000-00002 PMC2774214 15455579

[41] Bell S, Aggleton P, editors. Monitoring and evaluation in health and social development. London: Routledge; 2016.

[42] Rowe G, Horlick-Jones T, Walls J, et al Difficulties in evaluating public engagement initiatives: reflections on an evaluation of the UK GM Nation? public debate about transgenic crops. Public Understanding Sci. 2005;14:331–352.

[43] Fletcher G Addressing gender in impact evaluation. London: Overseas Development Institute; 2015.

[44] Charoenboon N, Haenssgen MJ, Warapikuptanun P, et al Translating antimicrobial resistance: a case study of context and consequences of antibiotic-related communication in three northern Thai villages. Palgrave Commun. 2019;5:23.

[45] Haenssgen MJ, Xayavong T, Charoenboon N, et al The consequences of AMR education and awareness raising: outputs, outcomes, and behavioural impacts of an antibiotic-related educational activity in Lao PDR. Antibiotics. 2018;7:95. 10.3390/antibiotics7040095 PMC6316454 30388824

[46] Haenssgen MJ, Charoenboon N, Do NTT, et al How context can impact clinical trials: a multi-country qualitative case study comparison of diagnostic biomarker test interventions. Trials. 2019;20:111. 30736818 10.1186/s13063-019-3215-9 PMC6368827

[47] Willis GB The practice of cross-cultural cognitive interviewing. Public Opin Q. 2015;79:359–395.

[48] Haenssgen MJ, Charoenboon N, Thavethanutthanawin P, et al Tales of Treatment: how local voices and public engagement activities can shape global health research and policy. Development Studies Association 2019 annual conference; 2019 619–21; Milton Keynes; 2019.

[49] WHO World antibiotic awareness week: 2016 campaign toolkit. Geneva: World Health Organization; 2016.

[50] WHO Global action plan on antimicrobial resistance. Geneva: World Health Organization; 2015. 10.7196/samj.9644 26242647

[51] Public Health England UK case of Neisseria gonorrhoeae with high-level resistance to azithromycin and resistance to ceftriaxone acquired abroad. Health Prot Report Adv Access Rep. 2018;12:11.

[52] Lim C, Takahashi E, Hongsuwan M, et al Epidemiology and burden of multidrug-resistant bacterial infection in a developing country. eLife. 2016;5:e18082. 27599374 10.7554/eLife.18082 PMC5030096

[53] Sumpradit N, Wongkongkathep S, Poonpolsup S, et al New chapter in tackling antimicrobial resistance in Thailand. BMJ. 2017;358 DOI:10.1136/bmj.j2423. PMC5582296 28874352

[54] Ministry of Health Health sector reform: strategy and framework till 2025. Vientiane: The Lao People’s Democratic Republic; 2013.

[55] WHOWPRO Meeting on multisectoral action on antimicrobial resistance in Cambodia, Lao People’s Democratic Republic and Viet Nam, 18–20 January 2017, Hanoi, Viet Nam. Manila: World Health Organization Regional Office for the Western Pacific; 2017.

[56] Michie S, van Stralen MM, West R The behaviour change wheel: a new method for characterising and designing behaviour change interventions. Implement Sci. 2011;6:42. 21513547 10.1186/1748-5908-6-42 PMC3096582

